# Diacylglycerol Kinases as Emerging Potential Drug Targets for a Variety of Diseases: An Update

**DOI:** 10.3389/fcell.2016.00082

**Published:** 2016-08-17

**Authors:** Fumio Sakane, Satoru Mizuno, Suguru Komenoi

**Affiliations:** Department of Chemistry, Graduate School of Science, Chiba UniversityChiba, Japan

**Keywords:** diacylglycerol kinase, bipolar disorder, hypospadias, Parkinson's disease, inhibitor, cancer, anti-tumor immunity

## Abstract

Ten mammalian diacylglycerol kinase (DGK) isozymes (α–κ) have been identified to date. Our previous review noted that several DGK isozymes can serve as potential drug targets for cancer, epilepsy, autoimmunity, cardiac hypertrophy, hypertension and type II diabetes (Sakane et al., 2008). Since then, recent genome-wide association studies have implied several new possible relationships between DGK isozymes and diseases. For example, DGKθ and DGKκ have been suggested to be associated with susceptibility to Parkinson's disease and hypospadias, respectively. In addition, the DGKη gene has been repeatedly identified as a bipolar disorder (BPD) susceptibility gene. Intriguingly, we found that DGKη-knockout mice showed lithium (BPD remedy)-sensitive mania-like behaviors, suggesting that DGKη is one of key enzymes of the etiology of BPD. Because DGKs are potential drug targets for a wide variety of diseases, the development of DGK isozyme-specific inhibitors/activators has been eagerly awaited. Recently, we have identified DGKα-selective inhibitors. Because DGKα has both pro-tumoral and anti-immunogenic properties, the DGKα-selective inhibitors would simultaneously have anti-tumoral and pro-immunogenic (anti-tumor immunogenic) effects. Although the ten DGK isozymes are highly similar to each other, our current results have encouraged us to identify and develop specific inhibitors/activators against every DGK isozyme that can be effective regulators and drugs against a wide variety of physiological events and diseases.

## Introduction

Mammalian diacylglycerol kinase (DGK) represents a large enzyme family (Goto et al., 2006; Sakane et al., 2007; Mérida et al., 2008; Topham and Epand, 2009). To date, ten mammalian DGK isozymes, α (Sakane et al., 1990; Schaap et al., 1990), β (Goto and Kondo, 1993), γ (Goto et al., 1994; Kai et al., 1994), δ (Sakane et al., 1996), ε (Tang et al., 1996), ζ (Bunting et al., 1996; Goto and Kondo, 1996), η (Klauck et al., 1996), θ (Houssa et al., 1997), ι (Ding et al., 1998), and κ (Imai et al., 2005), have been identified. Moreover, several alternative splicing products—such as δ1 and δ2 (Sakane et al., 2002); η1–η3 (Murakami et al., 2003; Shionoya et al., 2015); ζ1 and ζ2 (Ding et al., 1997), and ι1–ι3 (Ito et al., 2004)—have also been found. These isozymes contain two or three characteristic protein kinase C (PKC)-like C1 domains (cysteine-rich, zinc finger structures) and the catalytic region in common and are subdivided into five groups, type I (α, β and γ), II (δ, η and κ), III (ε), IV (ζ and ι), and V (θ), according to their structural features (Goto et al., 2006; Sakane et al., 2007; Mérida et al., 2008; Topham and Epand, 2009). Each group is characterized by the subtype-specific functional domains, such as EF-hand motifs (type I), pleckstrin homology and sterile α motif domains (type II), ankyrin repeats (type IV), and ras-associating and pleckstrin homology domains (type V).

Our previous review (Sakane et al., 2008) showed that many interesting studies on DGK have brought DGK to the center stage of diverse biological events such as growth factor/cytokine-dependent cell proliferation and motility, seizure activity, immune responses, cardiovascular responses, and glucose metabolism. Therefore, from a medical point of view, DGK isoforms are implicated in the pathogenesis of a wide variety of diseases, for example, cancer, epilepsy, autoimmunity, cardiac hypertrophy, hypertension, and type II diabetes. Thus, DGKs have emerged as potential and attractive drug targets for curing these diseases.

Recent advances in genotyping technology have allowed for rapid genome-wide screening of common variants in large populations, launching a new era in the investigation of the genetic basis of complex diseases. DGK is no exception. Since our review was published (Sakane et al., 2008), additional interesting reports using genome-wide association studies (GWASs) have successively implied several new possible relationships between DGK isozymes and diseases. For example, DGKη (Baum et al., 2008; Ollila et al., 2009; Squassina et al., 2009; Weber et al., 2011; Zeng et al., 2011), DGKκ (van der Zanden et al., 2011; Carmichael et al., 2013), and DGKθ (Pankratz et al., 2009; Simón-Sánchez et al., 2011) have been suggested to be associated with susceptibility to bipolar disorder (BPD), hypospadias, and Parkinson's disease, respectively.

Among these isozymes, based on the results obtained for the GWASs of *DGKH* (DGKη gene), we recently investigated the relationship between DGKη and BPD. For this purpose, we generated DGKη-knockout (KO) mice and used these mice to perform behavioral and pharmacological tests. Intriguingly, we found that DGKη-knockout mice showed lithium (BPD remedy)-sensitive mania-like behaviors, suggesting that DGKη is one of key enzymes of the pathogenesis of BPD (Isozaki et al., 2016).

As mentioned in our previous review (Sakane et al., 2008), the development of DGK isozyme-specific inhibitors/activators is important both for fundamental research and for developing therapeutic strategies to treat a wide variety of pathological disorders. However, there was no available DGK isozyme-specific inhibitor/activator until recently. We have recently identified DGKα-selective inhibitors using a newly established high-throughput screening method (Sato et al., 2013). Because DGKα has both pro-tumoral and anti-immunogenic properties, the DGKα-selective inhibitors would simultaneously have anti-tumoral and pro-immunogenic (anti-tumor immunogenic) effects.

This mini review will focus primarily on the two abovementioned topics, recent GWASs and the development of DGK isozyme-specific inhibitors.

## GWAS—new possible relationships between DGK isozymes and diseases

### DGKη

BPD is a highly heritable neuropsychiatric illness characterized by recurrent episodes of depression and mania or hypomania and affects up to 4% of the adult population worldwide (Bauer and Pfennig, 2005; Merikangas et al., 2007). Approximately 20% of the patients die of suicide (Kilbane et al., 2009). Recent GWASs of BPD have proposed novel genetic candidates, including *DGKH*, which encodes DGKη. Baum et al. for the first time, reported a strong association between BPD and three SNPs (rs9315885, rs1012053, and rs1170191) located in the first intron of *DGKH* by a GWAS in two independent samples of European origin (Baum et al., 2008; Table 1). Next, SNP rs9315885 was demonstrated to be associated with BPD in a Finnish family cohort (Ollila et al., 2009). In addition, six SNPs in *DGKH* including rs1170191 were associated with BPD in a German sample as well (Weber et al., 2011). Moreover, an association of *DGKH* with BPD has also been found in Sardinian (Squassina et al., 2009) and Chinese (Zeng et al., 2011) samples at the haplotype level. In addition, another study showed that BPD samples displayed significantly increased *DGKH* gene expression levels (25% higher than in controls; Moya et al., 2010). These data imply that mutations of the *DGKH* gene are involved in BPD. However, other studies have not confirmed this association (Sklar et al., 2008; Tesli et al., 2009; Yosifova et al., 2009). Moreover, GWAS itself does not directly indicate a relationship between SNPs and diseases. Therefore, it has been difficult to definitively conclude whether *DGKH* is related to BPD.

**Table 1:** **Summary of disease-associated SNPs of *DGK*η, κ, θ, γ, δ, and ι**.

**SNP name**	**Allele**	**Location**	**Gene**	**Disease/medical condition**	**References**
rs9315885	T	13q14.11	DGKη (Intron 1)	BPD	Baum et al., 2008; Ollila et al., 2009
rs1012053	A	13q14.11	DGKη (Intron 1)	BPD	Baum et al., 2008
rs1170191	C/A	13q14.11	DGKη (Intron 1)	BPD	Baum et al., 2008
				UPD	Weber et al., 2011
rs1170169	G	13q14.11	DGKη (Intron 1)	BPD	Weber et al., 2011
				UPD	Weber et al., 2011
				ADHD	Weber et al., 2011
rs2148004	G	13q14.11	DGKη (Intron 1)	UPD	Weber et al., 2011
rs994856	G	13q14.11	DGKη (Intron 3)	BPD	Weber et al., 2011
				UPD	Weber et al., 2011
				ADHD	Weber et al., 2011
rs9525580	A	13q14.11	DGKη (Intron 3)	BPD	Weber et al., 2011
				UPD	Weber et al., 2011
				ADHD	Weber et al., 2011
rs9525584	C	13q14.11	DGKη (Intron 7)	BPD	Weber et al., 2011
				UPD	Weber et al., 2011
rs1170101	G	13q14.11	DGKη (Intron 20)	BPD	Weber et al., 2011
				UPD	Weber et al., 2011
rs347405	C	13q14.11	DGKη (Intron 26)	ADHD	Weber et al., 2011
rs2122246	G	13q14.11	DGKη (Intron 14)	BPD	Zeng et al., 2011
rs1170099	A	13q14.11	DGKη (Intron 20)	SCZ	Zeng et al., 2011
rs1934179	A/G	Xp11.22	DGKκ (Intron 1)	Hypospadias	van der Zanden et al., 2011; Carmichael et al., 2013
rs7063116	A	Xp11.22	DGKκ (5′ upstream)	Hypospadias	van der Zanden et al., 2011; Carmichael et al., 2013
rs5961179	G	Xp11.22	DGKκ (Exon 15, synonymous codon)	Hypospadias	Carmichael et al., 2013
rs7882950	T	Xp11.22	DGKκ (Intron 14)	Hypospadias	Carmichael et al., 2013
rs12556919	T	Xp11.22	DGKκ (Intron 13)	Hypospadias	Carmichael et al., 2013
rs17003341	T	Xp11.22	DGKκ (Intron 10)	Hypospadias	Carmichael et al., 2013
rs1934190	G	Xp11.22	DGKκ (Intron 8)	Hypospadias	Carmichael et al., 2013
rs4143304	T	Xp11.22	DGKκ (Exon 6, synonymous codon)	Hypospadias	Carmichael et al., 2013
rs1934188	T	Xp11.22	DGKκ (Intron 4)	Hypospadias	Carmichael et al., 2013
rs17328236	G	Xp11.22	DGKκ (Intron 1)	Hypospadias	Carmichael et al., 2013
rs9969978	C	Xp11.22	DGKκ (Intron 1)	Hypospadias	Carmichael et al., 2013
rs1934183	T	Xp11.22	DGKκ (Intron 1)	Hypospadias	Carmichael et al., 2013
rs6614511	T	Xp11.22	DGKκ (Intron 1)	Hypospadias	Carmichael et al., 2013
rs5961183	C	Xp11.22	DGKκ (Intron 1)	Hypospadias	Carmichael et al., 2013
rs7876567	T	Xp11.22	DGKκ (Intron 1)	Hypospadias	Carmichael et al., 2013
rs1564282	T/A	4p16.3	DGKθ (3′ downstream)	Parkinson's disease	Pankratz et al., 2009
rs11248060	T/A	4p16.3	DGKθ (Intron 2)	Parkinson's disease	Pankratz et al., 2009
rs7647305	C	3q27.2	DGKγ (3′ downstream)	BMI	Melén et al., 2010
rs6798931	G/C	3q27.2	DGKγ (Intron 19)	BMI	Melén et al., 2010
rs11706414	T/A	3q27.2	DGKγ (3′ downstream)	Asthma	Melén et al., 2010
rs888383	C/G	3q27.2	DGKγ (Intron 19)	Asthma	Melén et al., 2010
rs1550532	C	2q37.1	DGKδ (Intron 1)	Bone density	O'Seaghdha et al., 2013
rs161339	G	7q32.3	DGKι (3′ downstream)	Obesity/BMI	Laramie et al., 2009

*BPD, bipolar disorder; UPD, unipolar depression; ADHD, attention deficit hyperactivity disorder; SCZ, schizophrenia; BMI, body mass index*.

All of the SNPs in *DGKH* that are implicated in the etiology of BPD by GWASs are located in introns and 3′-flank region (Table 1). For example, the SNPs rs9315885 and rs1170191, which are identified in multiple independent reports (Baum et al., 2008; Ollila et al., 2009; Weber et al., 2011), are located in the first intron of *DGKH*. Therefore, it is likely that the SNPs lead to dysregulation of the expression and generation of splice variants of DGKη, which probably cause BPD.

DGKη is known to be most abundantly expressed in the brain (Klauck et al., 1996; Usuki et al., 2015). Interestingly, the expression of DGKη increased between 1 and 4 weeks after birth, which coincides with synapse formation in the brain (Usuki et al., 2015). Moreover, a substantial amount of DGKη was detected in layers II–VI of the cerebral cortex; in the CA1, CA2, and dentate gyrus regions of the hippocampus; in the mitral cell and glomerular layer of the olfactory bulb; and in the Purkinje cells in the cerebellum of one—to 32-week-old mice (Usuki et al., 2015).

To test the association between DGKη and BPD, DGKη-KO mice are required. However, the generation of DGKη-KO mice has not been accomplished until recently. In our recent study, we succeeded in generating DGKη-KO mice, and performed a comprehensive behavioral analysis of the mice (Isozaki et al., 2016) to investigate the role of DGKη in higher brain functions and the relationship between this isozyme and BPD. DGKη-KO mice exhibited increased open field activity (the frequency of behavioral switching hyperactivity), increased open field center time/frequency (antianxiety), increased open arm time/frequency in elevated plus maze (antianxiety), and increased antidepressant-like behavior (Isozaki et al., 2016). Moreover, these phenotypes were sensitive to a BPD remedy, lithium. The behavioral profile (hyperactivity, lower anxiety, lower depressive states, and cognitive impairment) of DGKη-KO mice is similar in behavioral dimensions to BPD patients in the manic state (Martinowich et al., 2009), including the disappearance of the phenotypes upon lithium treatment. These lithium-sensitive phenotypes have been commonly observed in representative BPD model mice, such as neurocan-KO (Miró et al., 2012), clock-KO (Roybal et al., 2007), glutamate receptor 6-KO (Shaltiel et al., 2008), DGKβ-KO (Kakefuda et al., 2010; Shirai et al., 2010), and glycogen synthase kinase 3β-transgenic (Spittaels et al., 2000; Prickaerts et al., 2006) mice. Therefore, these findings strongly suggest that DGKη is one of the key enzymes related to BPD pathogenesis and support the GWAS results. The lack of availability of suitable animal models of mania has been one of the greatest impediments in the field. Our results indicate that the DGKη-KO mice would represent a bona fide model of human BPD with mania. Therefore, it is likely that these mice are particularly useful for studying the pathophysiology of mania. Moreover, DGKη-specific inhibitors can be good remedies for BPD patients in the depressive state.

DGKη has also been found to be associated with attention deficit hyperactivity disorder (ADHD) by GWAS (Weber et al., 2011). Moreover, mania-like behaviors are similar to ADHD symptoms. Therefore, DGKη-KO mice could also represent a model for ADHD, and there may be a possible link between DGKη and ADHD in addition to BPD (Table 1). GWASs have also implied that DGKη is associated with unipolar depression (Weber et al., 2011), and schizophrenia (Zeng et al., 2011). It is also interesting to investigate the relationship between DGKη and unipolar depression/schizophrenia. DGKη may commonly play pivotal roles in the pathology of these four psychoses.

DGKη-KO mice showed impairment in glycogen synthase kinase 3β signaling (Isozaki et al., 2016), which is closely related to BPD (Spittaels et al., 2000; Prickaerts et al., 2006). However, it is still unclear how DGKη is involved in the etiology of BPD. Phosphatidylinositol turnover has been hypothesized to play an important role in the mechanism of action of lithium (Martinowich et al., 2009). DGK is one of the components of phosphatidylinositol turnover (Goto et al., 2006; Sakane et al., 2007; Mérida et al., 2008; Topham and Epand, 2009). Moreover, we recently found that the pleckstrin homology domain of DGKη is selectively and strongly bound to phosphatidylinositol 4,5-bisphosphate, a product of phosphatidylinositol turnover (Kume et al., 2016). We also revealed that DGKη is a unique enzyme with high affinity for DG (Komenoi et al., 2015). In addition, DGKη is a positive regulator of the epidermal growth factor receptor/Raf/MEK/ERK pathway (Yasuda et al., 2009), which drives phosphatidylinositol turnover and is related to BPD (Sklar et al., 2008). It will be interesting to determine what role DGKη plays in the phosphatidylinositol turnover-related, lithium-sensitive molecular mechanisms of BPD pathogenesis.

### DGKκ

Hypospadias is a common congenital hypoplasia of the penis, affecting ~1 in 750 births in Europe. It is believed that hypospadias is caused by sex hormonal disturbances. In fact, genetic polymorphisms in endocrine-related genes such as estrogen receptors have been associated with hypospadias (Ban et al., 2008). To further identify the genetic variants in hypospadias, van der Zanden et al. performed the first GWAS using European samples of anterior or middle hypospadias patients and found that two SNPs, rs1934179 and rs7063116, in *DGK*κ, which mapped to Xp11.22 and encodes DGKκ, exhibited a significant association (van der Zanden et al., 2011; Table 1). The authors also found SNPs in *DGK*κ in additional Dutch and Swedish cohorts of anterior or middle hypospadias cases. Carmichael et al. confirmed that *DGK*κ variants are associated with hypospadias in a more racially/ethnically diverse study population of California births (Carmichael et al., 2013). In addition to rs1934179 and rs7063116, several other SNPs in *DGK*κ are associated with the disease. DGKκ mRNA is most abundant in the testis and placenta (Imai et al., 2005), and the study of van der Zanden et al. showed that expression of *DGK*κ was lower in preputial tissues in carriers of the risk allele rs1934179 (van der Zanden et al., 2011). These results indicate that *DGK*κ is a major risk gene for hypospadias.

### DGKθ

Parkinson's disease (PD) is a second most common chronic neurodegenerative disease with a cumulative prevalence of greater than one per thousand people (Kuopio et al., 1999). Mutations in five genes have been identified to influence PD risk in fewer than 5% of those with PD (Pankratz and Foroud, 2007). Three genes, *PARK2* (*parkin*), *PARK7* (*DJ1*), and *PINK1*, are typically transmitted with autosomal recessive inheritance and two, *SNCA* and *LRRK2*, are inherited in an autosomal dominant fashion. Mutations in all but *LRRK2* are typically found in early onset PD.

In addition to those five genes, two SNPs, rs1564282 and rs11248060, in the *GAK* (cyclin G associated kinase, a cell cycle regulator)*/DGKQ* (DGKθ) region were repeatedly reported to be associated with PD by Pankratz et al. (2009), and Simón-Sánchez et al. (2011) (Table 1). DGKθ is abundantly expressed in the brain (Houssa et al., 1997). Thus, these data suggest the identification of new susceptibility alleles for PD in the *GAK/DGKQ* region.

### Other DGK isozymes

genome-wide association studies have suggested that several other DGK isozymes are associated with diseases and medical conditions as follows: DGKγ: asthma (rs11706414, s888383) and obesity (rs7647305, rs6798931) in children (Melén et al., 2010); DGKδ (rs1550532): bone mineral density (O'Seaghdha et al., 2013); and DGKι (rs161339): obesity/body mass index (Laramie et al., 2009; Table 1).

## Specific inhibitors for DGK isozymes

DGKα (Sakane et al., 1990; Schaap et al., 1990) is highly expressed in hepatocellular carcinoma and melanoma cells (Yanagisawa et al., 2007; Takeishi et al., 2012). DGKα expression is involved in hepatocellular carcinoma progression and is a positive regulator of the proliferative activity of hepatocellular carcinoma through the Ras/Raf/MEK/ERK pathway (Takeishi et al., 2012). In melanoma cells, DGKα positively regulates tumor necrosis factor-α-dependent nuclear factor-κB (p65) activation via the PKC ζ-mediated Ser311 phosphorylation of p65 (Kai et al., 2009). The growth of colon and breast cancer cell lines was significantly inhibited by DGKα-siRNA and R59949 (Torres-Ayuso et al., 2014). The DGKα/atypical PKC/β1 integrin signaling pathway is essential for matrix invasion of breast carcinoma cells (Rainero et al., 2014). Therefore, the suppression of DGKα activity is expected to inhibit the progression of these cancers. On the other hand, DGKα is abundantly expressed in T lymphocytes, where it facilitates the non-responsive state known as anergy (Olenchock et al., 2006; Zha et al., 2006). Anergy induction in T cells represents the main mechanism by which advanced tumors avoid immune action. Therefore, if a DGKα-selective inhibitor is identified and developed, it would reversely attenuate cancer cell proliferation and simultaneously activate T cell function and can be a dual effective compound.

We started the “Dual effective DGKα-selective inhibitor project” in 2009. To develop highly effective and DGKα-selective inhibitors, a system for high-throughput screening is required; however, the conventional DGK assay is quite laborious and requires technical skill. For example, the conventional assay requires the use of a radioisotope ([γ-^32^P]ATP) and the manipulation of thin-layer chromatography with multiple extraction steps. We recently established a simple DGK assay (Sato et al., 2013) that is useful for constructing a high-throughput screening system for detecting DGK inhibitors from chemical compound libraries.

We screened a library containing core 9600 compounds (Drug Discovery Initiative, The University of Tokyo) using a high-throughput chemiluminescence-based assay. We obtained several compounds that inhibited the α-isozyme of DGK. Among the compounds, CU-3, 5-[(2E)-3-(2-furyl)prop-2-enylidene]-3-[(phenylsulfonyl)amino]-2-thioxo-1,3-thiazolidin-4-one was identified as a potent and selective inhibitor against the DGKα (Liu et al., 2016). Compared with commercially available DGK inhibitors, such as R59022 and R59949 (Sato et al., 2013), CU-3 exhibited higher efficiency and selectivity against DGKα. The IC_50_ value of CU-3 (0.6 μM) was markedly lower than the values of R59022 and R59949 (~25 and 18 μM, respectively; Sato et al., 2013). R59022 and R59949 only semi-selectively inhibited type I, III and V DGKs α, ε, and θ, and type I and II DGKs α, γ, δ, and κ, respectively (Sato et al., 2013). However, the IC_50_ value of CU-3 for DGKα was at least ~12 times lower than the values for other DGK isozymes. Therefore, this study is the first report of a highly α-isozyme selective inhibitor. The target of CU-3 is the catalytic domain of DGKα, and CU-3 competitively reduced the affinity of DGKα for ATP but not diacylglycerol or phosphatidylserine, strongly suggesting that CU-3 competes with ATP binding.

CU-3 induced apoptosis in HepG2 hepatocellular carcinoma and HeLa cervical cancer cells (Liu et al., 2016). Supporting our results, Torres-Ayuso et al. (Torres-Ayuso et al., 2014) also demonstrated that the growth of colon and breast cancer cell lines was significantly inhibited by DGKα-siRNA and R59949. In addition, Dominguez et al. reported that DGKα-siRNA and R59022 negatively affected the proliferation of glioblastoma, melanoma, breast cancer, and cervical cancer cells (Dominguez et al., 2013). The authors also observed that in marked contrast to cancer cells, R59022 did not weaken the growth of non-cancerous astrocytes and fibroblasts (Dominguez et al., 2013). CU-3 also failed to increase the caspase 3/7 activity of the non-cancer-derived COS-7 cells. These findings suggest that CU-3 selectively induces apoptosis.

In addition to the induction of cancer cell apoptosis, we found that CU-3 promoted IL-2 production, which is one of the indicators of T cell activation. Because inactivation (anergy induction) of T cells is the main mechanism by which advanced tumors to avoid immune action, it is expected that CU-3 is able to activate cancer immunity.

**Figure 1:**
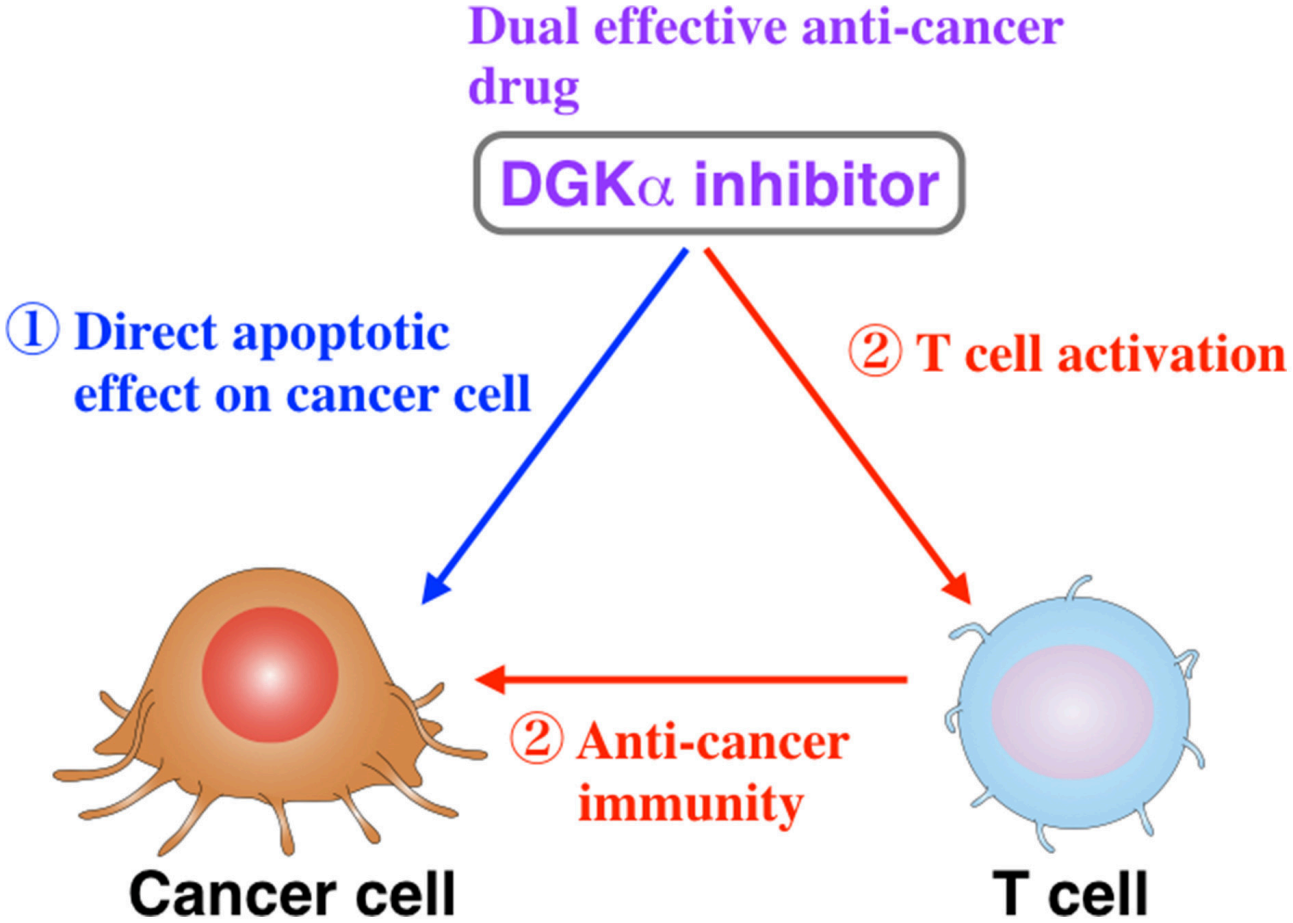
**A DGKα-selective inhibitor would directly attenuate cancer cell proliferation and simultaneously activate T cell function, which includes anti-tumor immunogenic activity (Liu et al., 2016)**.

General anti-cancer drugs inhibit the proliferation and function of both cancer and bone marrow cells (Chabner and Roberts, 2005; Pérez-Herrero and Fernández-Medarde, 2015). Therefore, they induce not only the attenuation of cancer cell proliferation but also bone marrow suppression/myelosuppression, which is one of the most commonly observed side-effects of anti-cancer drugs. However, there is no drug that has both pro-tumoral and anti-immunogenic effects. The DGKα-selective inhibitor would simultaneously have anti-tumoral and pro-immunogenic effects (Figure 1). Therefore, in addition to the direct effects on apoptosis induction in cancer cells, CU-3 can indirectly induce the death of cancer cells through activation of the immune system. Moreover, CU-3 can be an effective tool for biological science concerning cancer and immunity.

CU-3 still does not have sufficient isozyme selectivity and efficiency as an excellent inhibitor. Moreover, comprehensive studies where other kinase groups are tested have not been performed. Further refinement of CU-3 and/or identification/development of new candidates using larger chemical compound libraries are required. Finally, our current results encourage us to identify and develop specific inhibitors/activators against every DGK isozyme that can be effective regulators and drugs against a wide variety of physiological events and diseases, although the ten DGK isozymes are highly similar to each other.

## Author contributions

All authors listed, have made substantial, direct and intellectual contribution to the work, and approved it for publication.

### Conflict of interest statement

The authors declare that the research was conducted in the absence of any commercial or financial relationships that could be construed as a potential conflict of interest.
